# Draft Genome Sequences of 43 *Lactobacillus* Strains from the Species *L. curvatus*, *L. fermentum*, *L. paracasei*, *L. plantarum*, *L. rhamnosus*, and *L. sakei*, Isolated from Food Products

**DOI:** 10.1128/genomeA.00632-17

**Published:** 2017-07-27

**Authors:** Raffael C. Inglin, Leo Meile, Marc J. A. Stevens

**Affiliations:** Laboratory of Food Biotechnology, Institute of Food, Nutrition and Health, ETH Zurich, Zurich, Switzerland

## Abstract

The genome sequences of 43 *Lactobacillus* strains from the species *L. curvatus*, *L. fermentum*, *L. paracasei*, *L. plantarum*, *L. rhamnosus*, and *L. sakei* were determined using Illumina MiSeq.

## GENOME ANNOUNCEMENT

*Lactobacillus* strains have been isolated from a broad spectrum of food products, such as salami type sausages, meat, dairy products, sauerkraut, and fermented vegetables (1, 2). Lactobacilli are used as starter and protective cultures in industrial fermentations to control the fermentation process, extend the shelf-life of the fermented product, and increase its safety. In addition, some strains are marketed as probiotic and benefit the health of the consumer (3, 4). Here, the sequenced genomes of 4 *L. curvatus*, 1 *L. fermentum*, 3 *L. paracasei*, 28 *L. plantarum*, 1 *L. rhamnosus*, and 6 *L. sakei* strains are presented. These strains were selected from a phenotypic screening and exhibited an atypical phenotype, or were selected as potential protective cultures in a high-throughput screening assay (5). Genomic DNA was isolated by using a lysozyme-based cell wall digestion step and subsequently a Wizard genomic DNA purification kit (Promega, Dübendorf, Switzerland). The genomes were sequenced with Illumina MiSeq, pairwise reads of 150 bp, 30-fold coverage at the Functional Genomic Center Zurich (Zurich, Switzerland). Potential functions of predicted genes were automatically annotated using the NCBI Prokaryotic Genome Annotation Pipeline.

### Accession number(s).

Sequence and annotation data of the *Lactobacillus* strains are deposited as BioProject PRJNA343164 in the GenBank database and corresponding accession numbers listed in Table 1.

**TABLE 1:** Sequenced genomes of *Lactobacillus* in NCBI BioProject PRJNA343164

Strain	Accession no.	Genome size (Mb)	No. of contigs	% G+C	No. of CDS^a^
*Lactobacillus curvatus* RI-124	MKDR00000000	1.81	77	42.0	1,838
*Lactobacillus curvatus* RI-193	MKGD00000000	1.81	82	42.0	1,862
*Lactobacillus curvatus* RI-198	MKGC00000000	1.80	77	42.0	1,848
*Lactobacillus curvatus* RI-406	MKDG00000000	2.01	52	41.7	2,020
*Lactobacillus fermentum* RI-508	MKGE00000000	1.92	74	52.2	1,959
*Lactobacillus paracasei* RI-194	MKFZ00000000	3.06	86	46.3	3,197
*Lactobacillus paracasei* RI-195	MKGA00000000	3.03	125	46.3	3,170
*Lactobacillus paracasei* RI-210	MKFY00000000	3.06	58	46.1	3,164
*Lactobacillus plantarum* RI-011	MJHC00000000	3.17	33	44.6	3,063
*Lactobacillus plantarum* RI-012	MJHD00000000	3.22	101	44.4	3,175
*Lactobacillus plantarum* RI-048	MJHG00000000	3.19	94	44.5	3,150
*Lactobacillus plantarum* RI-086	MKDP00000000	3.08	90	44.6	3,003
*Lactobacillus plantarum* RI-123	MKDQ00000000	3.32	60	44.3	3,253
*Lactobacillus plantarum* RI-139	MKDS00000000	3.33	78	44.4	3,264
*Lactobacillus plantarum* RI-140	MKDT00000000	3.31	85	44.3	3,241
*Lactobacillus plantarum* RI-146	MKDU00000000	3.37	61	44.3	3,304
*Lactobacillus plantarum* RI-147	MKDV00000000	3.32	100	44.4	3,253
*Lactobacillus plantarum* RI-162	MJHH00000000	3.32	41	44.6	3,053
*Lactobacillus plantarum* RI-165	MJHI00000000	3.30	101	44.3	3,212
*Lactobacillus plantarum* RI-189	MJHJ00000000	3.10	56	44.5	3,012
*Lactobacillus plantarum* RI-190	MJHK00000000	3.10	58	44.5	2,996
*Lactobacillus plantarum* RI-208	MKFX00000000	3.14	82	44.5	3,042
*Lactobacillus plantarum* RI-266	MKDY00000000	3.47	71	44.2	3,412
*Lactobacillus plantarum* RI-405	MKDF00000000	3.32	72	44.3	3,273
*Lactobacillus plantarum* RI-408	MKDH00000000	3.07	123	44.6	2,988
*Lactobacillus plantarum* RI-422	MKDK00000000	3.33	55	44.3	3,274
*Lactobacillus plantarum* RI-505	MKDZ00000000	3.10	52	44.7	3,018
*Lactobacillus plantarum* RI-506	MKEA00000000	3.39	76	44.2	3,344
*Lactobacillus plantarum* RI-507	MKEB00000000	3.53	126	44.1	3,499
*Lactobacillus plantarum* RI-509	MKEC00000000	3.32	66	44.4	3,296
*Lactobacillus plantarum* RI-510	MKED00000000	3.37	110	44.2	3,353
*Lactobacillus plantarum* RI-511	MKEE00000000	3.33	105	44.2	3,300
*Lactobacillus plantarum* RI-512	MKEF00000000	3.30	151	44.3	3,284
*Lactobacillus plantarum* RI-513	MKEG00000000	3.28	71	44.4	3,199
*Lactobacillus plantarum* RI-514	MKEH00000000	3.23	61	44.5	3,134
*Lactobacillus plantarum* RI-515	MKGF00000000	3.32	94	44.4	3,258
*Lactobacillus rhamnosus* RI-004	MJHB00000000	2.92	72	46.6	2,993
*Lactobacillus sakei* RI-394	MKDC00000000	1.94	44	41.0	1,963
*Lactobacillus sakei* RI-403	MKDD00000000	2.00	29	41.0	2,032
*Lactobacillus sakei* RI-404	MKDE00000000	1.95	28	41.0	1,977
*Lactobacillus sakei* RI-409	MKGB00000000	1.99	67	40.9	2,022
*Lactobacillus sakei* RI-410	MKDI00000000	1.93	73	41.1	1,949
*Lactobacillus sakei* RI-412	MKDJ00000000	1.92	32	41.1	1,934

a CDS, coding sequences.
